# Correction: Zhu et al. HIF-1α-Overexpressing Mesenchymal Stem Cells Attenuate Colitis by Regulating M1-like Macrophages Polarization toward M2-like Macrophages. *Biomedicines* 2023, *11*, 825

**DOI:** 10.3390/biomedicines13081903

**Published:** 2025-08-05

**Authors:** Wenya Zhu, Qianqian Chen, Yi Li, Jun Wan, Jia Li, Shuai Tang

**Affiliations:** 1 Medical School of Chinese PLA, Beijing 100039, China; zhuwenya_1985@163.com; 2 Department of Geriatrics, The Sixth Medical Center, Chinese PLA General Hospital, Beijing 100048, China; 3 Department of Gastroenterology, The Second Medical Center & National Clinical Research Center for Geriatric Diseases, Chinese PLA General Hospital, Beijing 100039, China; juliazcx@163.com (J.L.); tangs218@163.com (S.T.); 4 Department of Gastroenterology, The First Medical Center, Chinese PLA General Hospital, Beijing 100039, China; liyi1986322@163.com

## Error in Figures

In the original publication [1], there was a mistake in Figures 4 and 6 as published. We mistakenly placed the F4/80 IF image of the HIF group in the TNBS group, inducing duplicates between the HIF group and TNBS group images. The corrected Figure 4 and Figure 6 appear below. The authors state that the scientific conclusions are unaffected. This correction was approved by the Academic Editor. The original publication has also been updated.

## Figures and Tables

**Figure 4:**
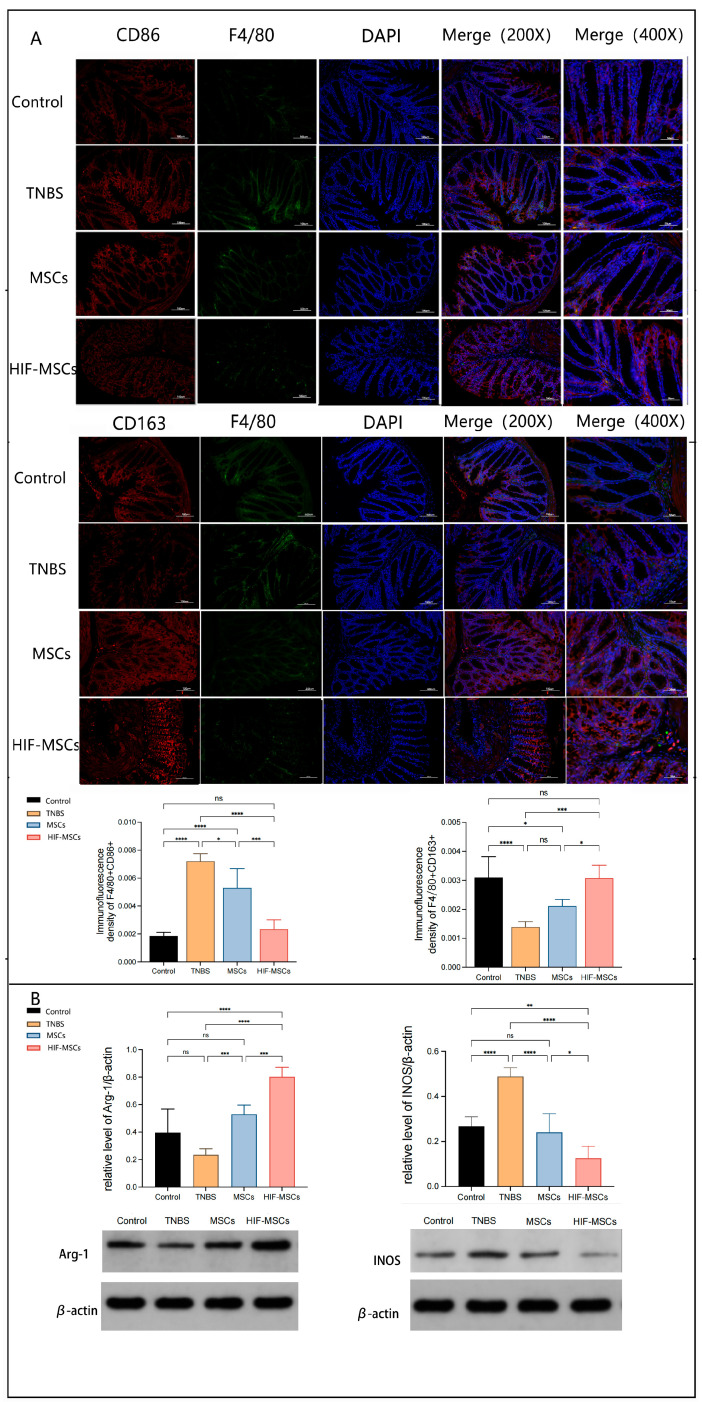
HIF-MSCs promoted M1 macrophage polarization toward M2 macrophages in vivo. (**A**). Immunofluorescence analysis was used to detect the expressions of F4/80+CD86+ and F4/80+CD163+ in colonic tissue, revealing that HIF-MSCs significantly decreased the relative expression ratio of F4/80+CD86+ and increased the relative expression ratio of F4/80+CD163+ compared with those promoted by PBS and MSCs (**** *p* < 0.0001, *** *p* < 0.001, * *p* < 0.05). (**B**). Western blotting analysis was used to detect the M2 characteristic Arg-1 and the M1 characteristic INOS. HIF-MSCs upregulated Arg-1 expression and downregulated INOS expression in colon tissue compared with PBS and MSCs (**** *p* < 0.0001, *** *p* < 0.01, ** *p* < 0.01, * *p* < 0.05). “ns” represents no significant difference.

**Figure 6:**
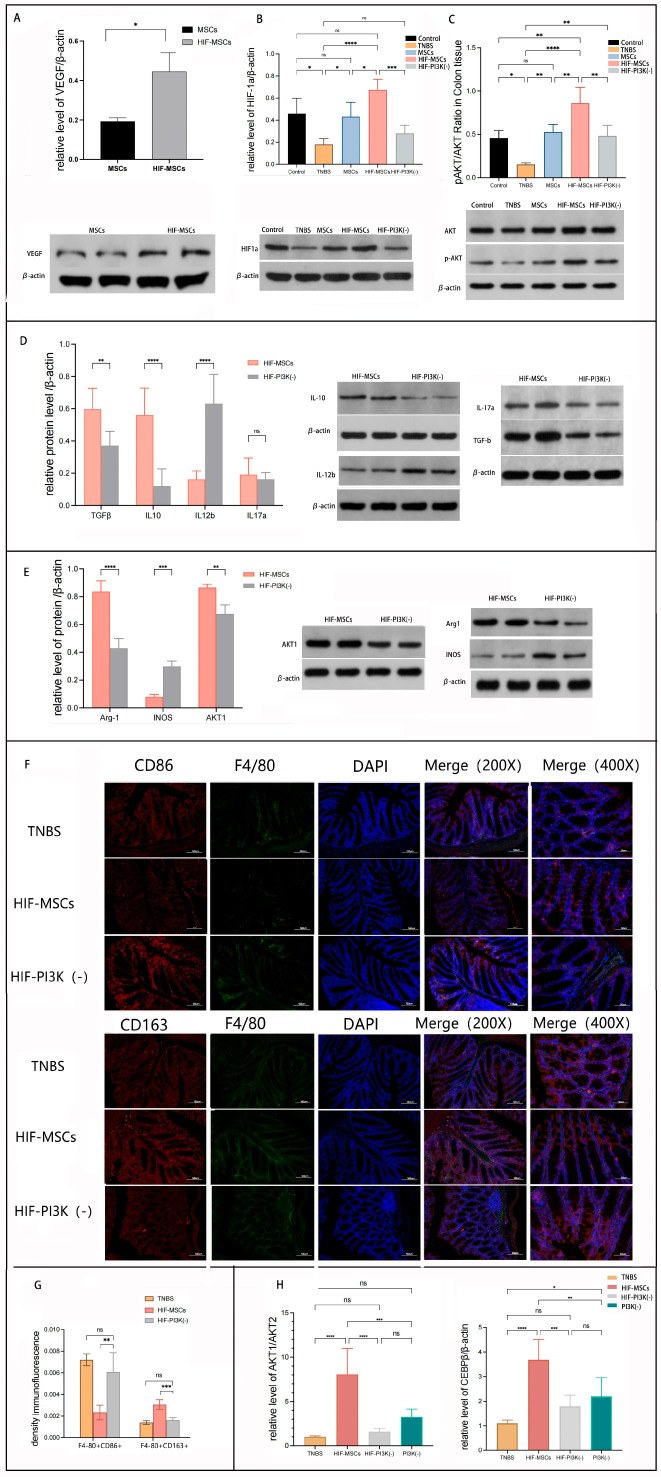
HIF-MSCs affected macrophage polarization through the PI3K-γ pathway. IPI549 was used to block the PI3K-γ pathway in mice and in induced M1-like macrophages. (**A**). Western blotting was used to detect VEGF in HIF-MSCs and MSCs. VEGF had a significantly higher expression in HIF-MSCs (* *p* < 0.05). (**B**,**C**). HIF-MSC treatment upregulated the expression of HIF-1α and *p*-AKT/AKT when compared with those of MSCs and PBS (**** *p* < 0.0001, *** *p* < 0.001, ** *p* < 0.01, * *p* < 0.05). PI3K-γ inhibition blocked the upregulatory effect of HIF-MSCs on HIF-1α and *p*-AKT/AKT (*** *p* < 0.001, ** *p* < 0.01). (**D**). PI3K-γ inhibition attenuated the regulatory effect of HIF-MSCs on inflammatory factors; there was a significant difference in IL-12b, TGF-β, and IL-10 expression between the HIF-MSC group and the HIF-MSC–PI3K-γ inhibition group (**** *p* < 0.0001, ** *p* < 0.01). (**E**). PI3K-γ inhibition decreased the expression of Arg-1 and AKT1 and increased that of INOS (**** *p* < 0.0001, *** *p* < 0.001, ** *p* < 0.01). (**F**,**G**). PI3K-γ inhibition decreased the relative expression ratio of F4/80+CD163+ and increased the relative expression ratio of F4/80+CD86+ (*** *p* < 0.001, ** *p*< 0.01) in colonic tissue compared with the HIF-MSC group. (**H**). qPCR was used to detect AKT1, AKT2, and C/EBPβ expression in macrophages. HIF-MSCs had upregulated AKT1/AKT2 and C/EBPβ expression; the effect was significantly blocked upon PI3K-γ initiation treatment (**** *p* < 0.0001, *** *p* < 0.001, ** *p* < 0.01, * *p* < 0.05). “ns” represents no significant difference.
