# Supplementary material for: HIF-1α-Overexpressing Mesenchymal Stem Cells Attenuate Colitis by Regulating M1-like Macrophages Polarization toward M2-like Macrophages
Source: Biomedicines. 2023 Mar 8;11(3):825. doi: 10.3390/biomedicines11030825 (PMC10045413; doi:10.3390/biomedicines11030825)
Supplement: Supplementary file 1 [file biomedicines-11-00825-s001.zip › biomedicines-2239573-supplementary.pdf]

Supplementary Materials

Table S1 HIF1A Gene amplification Primer Sequence

ID	Sequence (5'-3')
HIF1A-P1	AGGTCGACTCTAGAGGATCCCGCCACCATGGAGGGCGCCGGCGGCG
HIF1A-P2	TCCTTGTAGTCCATACCGTTAACTTGATCCAAAGCTCTGAG

Table S2 Primer Sequence

Gene	Primer	Sequence (5'-3')
GAPDH	Forward	TCAAGAAGGTGGTGAAGCAGG
	Reverse	TCAAAGGTGGAGGAGTGGGT
β-ACTIN	Forward	GCACAGAGCCTCGCCTT
	Reverse	GTTGTCGACGACGAGCG
HIF-1A	Forward	GTGGCGAAGATGGTCAAGTC
	Reverse	GGAGTGCCCTTGTTGAGGTGTT
TGF-β	Forward	TACAGCAACAATTCCTGGCGATACC
	Reverse	AATTTCCCCTCCACGGCTCAAC
IL-10	Forward	TTCACAGGGAAGAAATCG
	Reverse	ACCAAGACCCAGACATCA
IL-12b	Forward	TCACAAAGGAGGCGAGGTTCTA
	Reverse	CTCTGCTGCTTTTGACACTGAATG
IL-17A	Forward	GGATGCCCAAATTCTGAGGAC
	Reverse	ACTTTGCCTCCCAGATCACA
IL-23	Forward	TGGCTGTGCCTAGGAGTAGCA
	Reverse	TTCATCCTCTTCTTCTCTTAGTAGATTCATA
IL-6	Forward	AA TTTCCTCTGGTCTTCTGG
	Reverse	ACTCTGGCTTTGTCTTTCTTGTT
TNF-a	Forward	GCCAGGAGGGAGAACAGAAACTC
	Reverse	GGCCAGTGAGTGAAAGGGACA
LDHA	Forward	CCAACATGGCAGCCTTTTCC
	Reverse	TCACGTTACGCTGGACCA AA
CXCR-4	Forward	CCTCTACAGCAGCGT TCT
	Reverse	GTT TCC TTG GCC TTT GAC
VEGF	Forward	ACATTGGTCACTTCCAGAAACAC
	Reverse	TGGTTGGAACCGGCATCTTA

GAPHD	Forward	CTCTGCTCCTCCTGTTCGAC
	Reverse	GCGCCCAATACGACCAAATC
AKT1	Forward	GCACAAACGAGGGGAGTACATC
	Reverse	GCCATCATTCTTGAGGAGGAAGTAG
AKT2	Forward	TGTTAGCACTTCACACCCATTG
	Reverse	CCCCAACCAAACGAGTCCTA
C/CEBPβ	Forward	CCTGAGTAATCGCTTAAAGATGTTC
	Reverse	GTGTTCTTAATGCTTGAAACGGA
Arg-1	Forward	TGCCCTTTGCTGACATCCCTAAT
	Reverse	TTCTTCCGTTCTTCTTGACTTCTGC
CCR-7	Forward	CAGAGGAGCAGCAGTGAGCAAG
	Reverse	GGTGCGGATGATGACAAGGTAACAG
INOS	Forward	CAAGCTGAACTTGAGCGAGGA
	Reverse	TTTACTCAGTGCCAGAAGCTGGA

Figure S1. The adipogenic and osteogenic differentiation of HIF-MSCs.

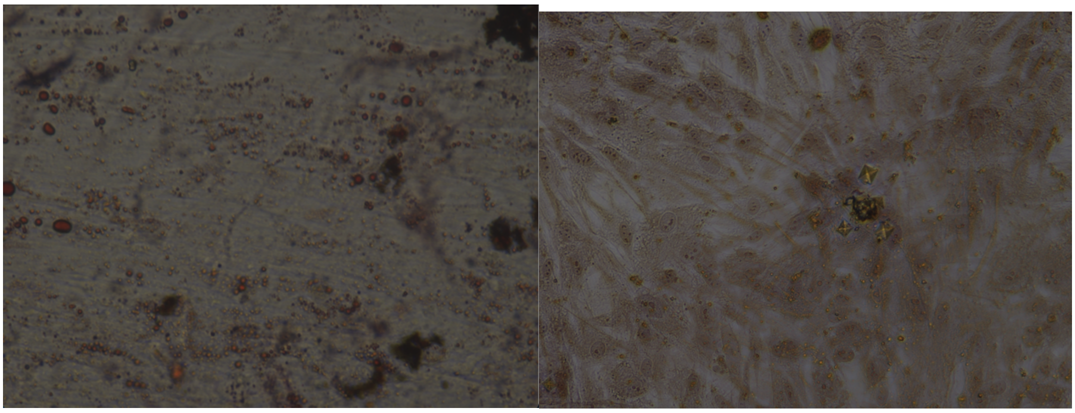

Figure S2. GFP-labeled MSCs and HIF-MSCs colonized in intestinal tissue 3 days after transplantation.

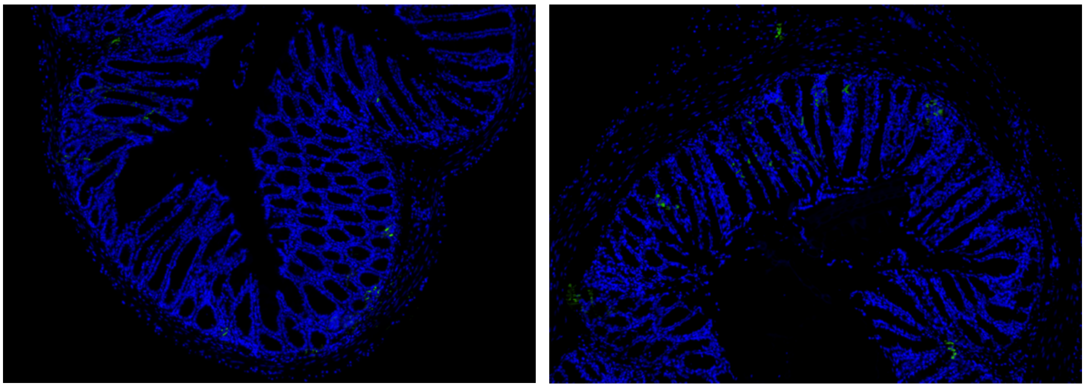

GFP-MSCs GFP-HIFMSCs
